# Copper-decorated core–shell structured ordered mesoporous containing cobalt ferrite nanoparticles as high-performance heterogeneous catalyst toward synthesis of tetrazole

**DOI:** 10.1038/s41598-023-42094-1

**Published:** 2023-09-13

**Authors:** Somayeh Molaei, Mohammad Ghadermazi

**Affiliations:** Department of Chemistry, Faculty of Science, University of Kurdistan, Sanandaj, Iran

**Keywords:** Catalysis, Green chemistry

## Abstract

The present work describes the synthesis of copper immobilization on CoFe_2_O_4_/MCM-41 as a catalyst, which is created by attaching copper and ligand (N-phenyl anthranilic acid (PA)) on the surface of CoFe_2_O_4_/MCM-41 (CoFe_2_O_4_/MCM-41/PA/Cu). The synthesized CoFe_2_O_4_/MCM-41 support and immobilized copper were identified by FTIR, TEM, VSM, SEM XRD, and nitrogen adsorption–desorption analysis. The results showed that MCM-41 silica was coated with magnetite nanoparticles and copper was successfully immobilized on this structure. The catalytic performance of synthesized catalyst was tested in the synthesis of tetrazole. It was shown that the solid catalyst exhibited a strong magnetic response and showed good catalytic activity in the synthesis of tetrazole. The catalytic test showed that copper supported on CoFe_2_O_4_/MCM-41 hybrid showed much better catalytic activity than copper supported on CoFe_2_O_4_, indicating that MCM-41 plays an important role in CoFe_2_O_4_/MCM-41 hybrid for the synthesis of tetrazole. Separation of the solid catalyst from the reaction mixture was easily performed by external magnetism without apparent mass loss. And the catalyst could be reused six times for the synthesis of heterogeneous tetrazole.

## Introduction

Metal nano oxides have attracted a lot of attention in recent years due to their unique properties, which include a large surface area. Ferrites are those magnetic materials whose main component is "iron oxide" and for this reason they have favorable magnetic properties^1,2^. Ferrites are ceramics that are considered as magnetic materials. Ferrites, like ferromagnetic materials, have structures in the magnetic domain and the residual ring. The crystal structure of spinel is one of the ternary structures of materials that have the general formula AB_2_O_4_, where A and B can be cations of different metals. In the spinel crystal lattice structure, divalent ions A (such as Co^2+^) are located in tetrahedral cavities and trivalent ions B (such as Fe^3+^) are located in octahedral cavities^3^. Cobalt ferrite has received much attention in recent years as a catalyst substrate^4,5^. By reducing the diameter of these magnetic particles to nano dimensions, the available external surface area for surface modification increases^6^. After surface modification, these particles are easily spread inside the solvent and create a stable diffusion. Silica is a very suitable coating for the modification of magnetic nanoparticles. Due to its richness in hydroxy functional groups for subsequent modifications, as well as its high thermal and mechanical stability^7^.

Mesoporous silicates due to having appropriate characteristics such as chemical and thermal stability, having the appropriate shape and degree of porosity, as well as having a surface that can specifically react with organic and inorganic groups, in the fields of protection of catalysis, removal of heavy metals from water and many other fields such as surface adsorption and chemical separation^8,9^. MCM-41 is an inorganic substance. The pore size of MCM-41 changes from 20 to 100 Å during synthesis and each gram of this material has an area of about 1000–1300 m^2^^10^. Due to the easier synthesis of MCM-41 compared to other substances, this substance has been used the most^11^. For easy separation and better use of MCM-41, it is better to cover it with magnetic compound. For effective applications of silicate mesoporous structures in many basic processes, it is necessary to modify the surface of these solids by functional groups. Silica mesoporous have many silanol groups on their surface. These surface groups can be converted into other groups by chemical reactions. By attaching different functional groups and organic structures on the surface, mesoporous surface properties such as hydrophilicity and hydrophobicity and the ability to bind to guest molecules can be controlled^12^. Modifying mesoporous materials gives various properties to these materials so that these materials find various applications in fields such as catalysis of chemical reactions, separation processes and making chemical sensors. The presence of free silanol groups on the surface of mesoporous silica materials shows that by functionalizing these porous silica materials, active and suitable sites can be created to perform reactions on these materials^13^. It is possible to prevent the separation of catalytically active species from the support surface by creating covalency. Incorporation of metal centers in the mesoporous silica materials is necessary to make the materials as potential catalysts.

Tetrazoles are acyclic compounds that consist of a five-membered ring containing four nitrogens and one carbon^14^. Due to having a low energy occupied molecular orbital (HOMO), tetrazoles resist the strongest oxidizing agents^15^. These compounds are acidic and their pK_a_ is similar to their corresponding carboxylic acids^16^. These compounds have various applications in the synthesis of other organic substances, pharmaceutical and biological industries as well as military industries^17^. Due to having high potential energy, low sensitivity to impact and friction, and having high explosion heat, tetrazoles have a good potential to be used in gas and explosives producers. These compounds are suitable substitutes for explosives due to their lower toxicity, less soot production and greater strength. Among the medicinal compounds containing tetrazole ring, we can mention antibacterial, antimicrobial, antiviral, antidiabetic, anti-schizophrenic, antihypertensive and cyclooxygenase-2 inhibitor^18–21^. Also, tetrazoles are used as propellants in rocket and jet engines in the aerospace industry^22,23^. Due to having several ring coordination centers and high ring electron density, tetrazoles are used in the synthesis of complex compounds and also as protective insulators for metals^24^. The synthesis of tetrazole ring is a vital step in organic and medicinal chemistry, and various methods have been provided for the synthesis of these compounds^25–27^. The results of the conversion of nitril to tetrazole over various heterogeneous and homogeneous catalysts are summarized in Table 1. The non-reusability of the homogeneous catalyst, the low tetrazole yields, and toxic reaction conditions hindered their use for the synthesis of tetrazole. Despite the verity of studies on the use of catalysts for the direct synthesis of tetrazole, there is no paper has focused on the use of CoFe_2_O_4_/MCM-41-based heterogenous catalysts for the synthesis of tetrazole. Therefore, it is highly desirable to progress an efficient catalyst for the selective synthesis of tetrazole under mild reaction conditions in a benign environmental solvent.

**Table 1:** Catalytic performance of different heterogeneous and homogeneous catalysts for conversion of nitrile to tetrazole via sodium azide.

Entry	Catalyst	T (°C)	Solvent	Substrate	Time (min)	Yield (%)	Ref
1	Fe_3_O_4_@MCM-41@Cu-P2C (20 mg)	120	PEG	4-Bromobenzonitrile	3 h	96	Nikoorazm and Erfani ^28^
				4-Chlorobenzonitrile	3 h	96	
				4-Nitrobenzonitrile	4 h	98	
				Benzonitrile	3.5 h	95	
2	CoFe_2_O_4_@glycine-Yb (70 mg)	120	PEG	4-Bromobenzonitrile	130	92	Tamoradi et al.^29^
				4-Chlorobenzonitrile	140	93	
				4-Nitrobenzonitrile	140	96	
				Benzonitrile	159	95	
3	Cu (II) immobilized on Fe_3_O_4_@SiO_2_@L-histidine (50 mg)	120	PEG	4-Bromobenzonitrile	9 h	89	Azadi et al.^30^
				4-Chlorobenzonitrile	240	95	
				4-Nitrobenzonitrile	24 h	98	
				Benzonitrile	180	90	
4	Fe_3_O_4_@SiO_2_/salen Cu (II) (0.4 mol %)	120	DMF	4-Bromobenzonitrile	6.5 h	88	Dehghani et al.^31^
				4-Chlorobenzonitrile	6 h	88	
				4-Nitrobenzonitrile	6 h	92	
				Benzonitrile	7 h	90	
5	Cu-TBA@biochar (0.78 mol %)	130	PEG	4-Bromobenzonitrile	680	95	Moradi et al.^32^
				4-Chlorobenzonitrile	140	89	
				4-Nitrobenzonitrile	300	88	
				Benzonitrile	420	98	
6	Cu (II)-DCC-CMK-3 (20 mg)	120	PEG	4-Bromobenzonitrile	180	91	Ghafouri-Nejad and Hajjami^33^
				4-Chlorobenzonitrile	65	95	
				4-Nitrobenzonitrile	70	92	
				Benzonitrile	90	96	
7	SO_3_H@MCM-41 (50 mg)	80	DMF	4-Bromobenzonitrile	120	85	Matloubi Moghaddam et al.^34^
				4-Chlorobenzonitrile	120	90	
				4-Nitrobenzonitrile	120	80	
				Benzonitrile	120	90	
8	Ni-cytosine@MCM-41 (0.1 mol%),	120	PEG	4-Hydroxybenzonitrile	30	97	Nikoorazm et al.^35^
				3-Chlorobenzonitrile	140	90	
				4-Nitrobenzonitrile	90	93	
				Benzonitrile	60	92	
9	PdCl_2_ (27.8 mg)	120	DMF	4-Bromobenzonitrile	8 h	79	Kant et al.^36^
				4-Chlorobenzonitrile	8 h	77	
				4-Methylbenzonitrile	10 h	75	
				Benzonitrile	8 h	81	
10	(NH_4_) Ce (NO_3_)_6_ (10 mmol %)	110	DMF	4-Bromobenzonitrile	6 h	98	Kumar et al.^37^
				4-Chlorobenzonitrile	6 h	95	
				4-Methylbenzonitrile	6 h	94	
				Benzonitrile	6 h	97	
11	Amberlyst-15 (23 mol %)	85	DMSO	4-Methylbenzonitrile	12 h	89	Shelkar et al.^38^
				4-Chlorobenzonitrile	12 h	93	
				4-Nitrobenzonitrile	12 h	84	
				Benzonitrile	12 h	91	
12	L-Cysteine@MCM-41(2.9 mol %)	100	PEG	4-Bromobenzonitrile	3.25 h	97	Nikoorazm et al.^39^
				4-Chorobenzonitrile	2 h	97	
				4-Nitrobenzonitrile	2.5 h	97	
				Benzonitrile	3 h	98	
13	Pd-SBT@MCM-41 (35 mg)	120	PEG	4-Bromobenzonitrile	14 g	87	Nikoorazm et al.^40^
				4-Chorobenzonitrile	8 h	91	
				4-Nitrobenzonitrile	4 h	92	
				Benzonitrile	8 h	96	
14	CoFe_2_O_4_/MCM-41/PA/Cu (70 mg)	80	H_2_O	4-Bromobenzonitrile	10	90	This work
				4-Chorobenzonitrile	10	84	
				4-Nitrobenzonitrile	20	87	
				Benzonitrile	20	91	

In this paper, we demonstrated that MCM-41/PA/Cu alloys deposited on CoFe_2_O_4_, with post-functionalization modification method and could be used for the synthesis of tetrazole in an aqueous medium, which was more suitable than other reported catalysts. A Comparison of methods in synthesis of heterogeneous catalysts are summarized in Table 2. The reason for the design of CoFe_2_O_4_/MCM-41/PA/Cu catalyst is as follows: (1) The high Lewis acidity of the catalyst can facilitate the adsorption of substrate. (2) Using magnetic properties for easier separation. (3) MCM-41 protects the magnetic cores from environmental factors, and therefore, recoverable magnetic nano catalysts can be used in relatively harsh reaction conditions (strong acidic conditions, hard oxide etc.). (4) Catalyst aggregation and clumping are effectively prevented by the outer layer of porous silica. (5) Porous silica creates a passageway for substrates to enter and products to exit. (6) Using pores of MCM-41 to attach more active center. (7) The synthesis method was very convenient and safe. To elucidate the significant activity of CoFe_2_O_4_/MCM-41/PA/Cu catalyst, CoFe_2_O_4_/PA/Cu catalyst was prepared, and catalytic performance was compared with that of the CoFe_2_O_4_/MCM-41/PA/Cu catalyst.

**Table 2:** A Comparison of methods in synthesis of heterogeneous catalysts.

Catalyst	Methods	Simplicity	Efficacy	Surface area (m^2^/g)	Ref
STA/MCM-48	Hydrothermal and wet-impregnation	Simple	Comparable conversion	428.3	Şimşek and Şahin^41^
Pt–Au/CeO_2_	Deposition Precipitation (DP)	Quite complicated	High activity	175.1	Hong et al.^42^
CoPd/C heterogeneous catalysts	Charge-Enhanced Dry Impregnation (CEDI)	Simple	High metal surface-to-bulk ratios	280	Barnes et al.^43^
Pt/SiO_2_	Strong Electrostatic Adsorption (SEA)	Quite simple	Pt loading (2.68 wt%)	200	Miller et al.^44^
CoFe_2_O_4_/MCM-41/PA/Cu	Post-functionalization modification	Simple	High activity	437.2	This work

## Material and methods

**Figure 1:**
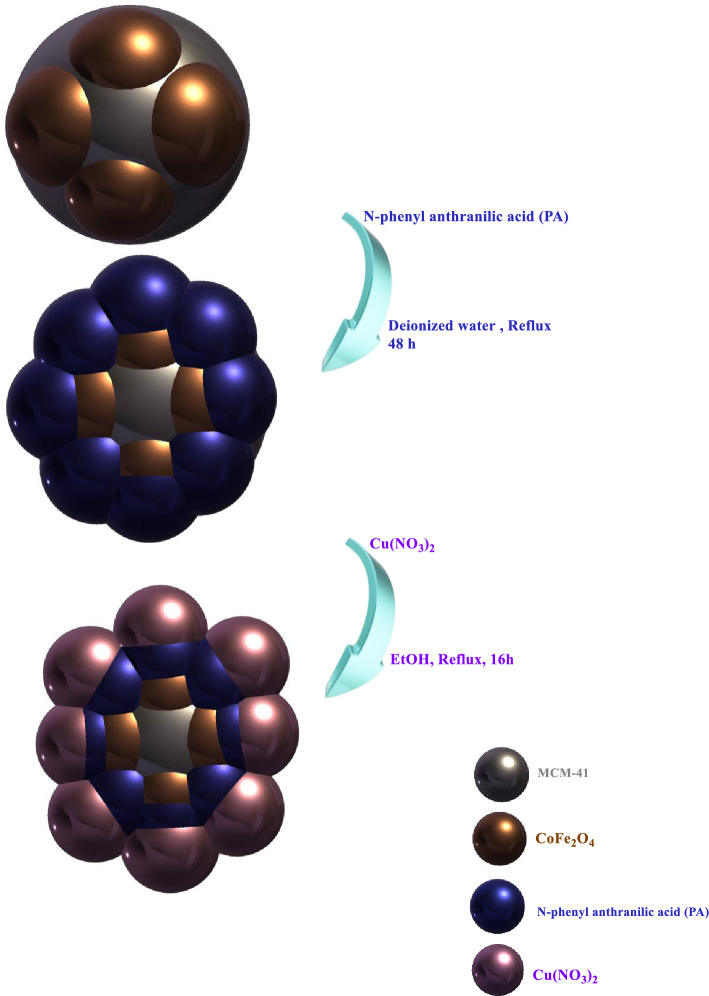
Schematic illustration of the synthesis of the CF/M/PA/C denotes nanoalloy with CF (CoFe_2_O_4_), M (MCM-41), N-phenyl anthranilic acid (PA), and Cu (C).

As shown in Fig. 1, the CoFe_2_O_4_/MCM-41/PA/Cu catalyst (CF/M/PA/C denotes nanoalloy with CF (CoFe_2_O_4_), M (MCM-41), N-phenyl anthranilic acid (PA), and Cu (C)) was successfully fabricated. Catalyst preparation, and product analysis are given in the Supporting Information.

## Results and discussion

### Catalyst characterization

#### XRD analysis

**Figure 2:**
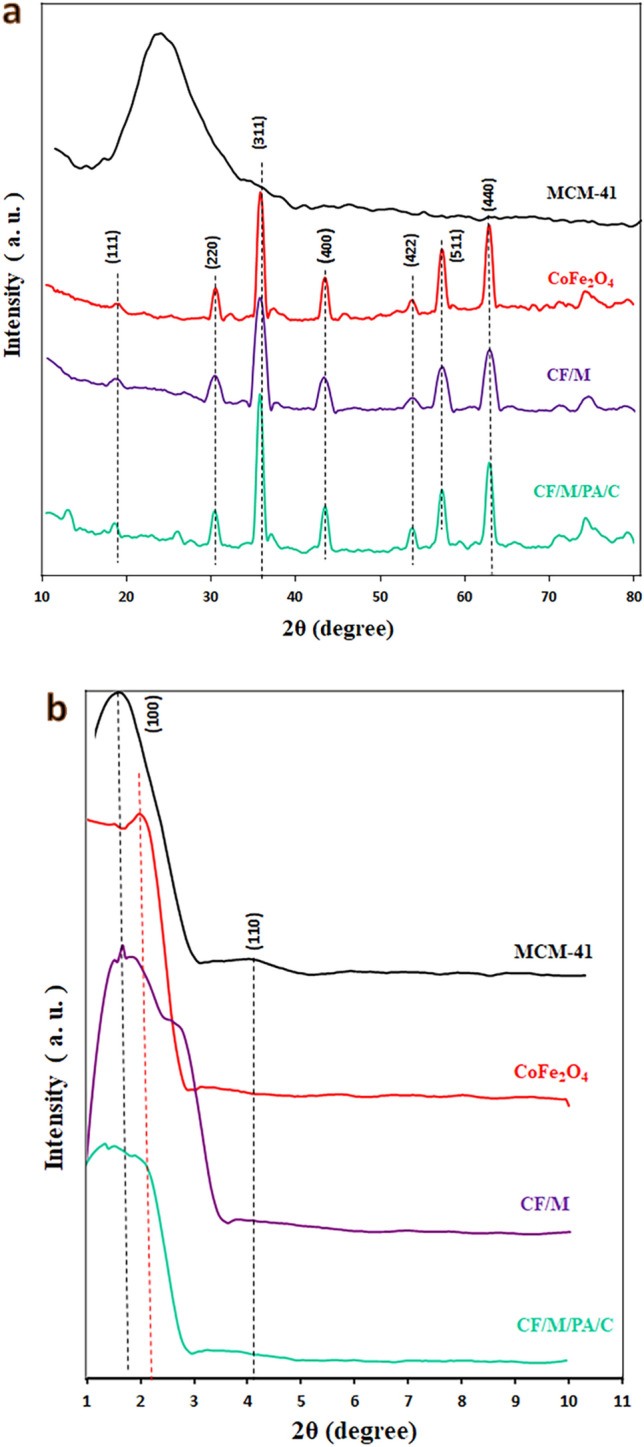
XRD patterns (**a**) normal and (**b**) low angle of the MCM-41, CoFe_2_O_4_, CF/M, and CF/M/PA/C denotes nanoalloy with CF (CoFe_2_O_4_), M (MCM-41), N-phenyl anthranilic acid (PA), and Cu (C).

Figure 2 presents the XRD patterns of the MCM-41, CoFe_2_O_4_, CoFe_2_O_4_/MCM-41 and CoFe_2_O_4_/MCM-41/PA/Cu catalyst. The XRD profiles of all samples show the characteristic diffraction peaks at round 18.1, 31.2, 36.5, 44.0, 54.2, 57.9, and 63.1° assigned to the (111), (220), (311), (400), (422), (511), and (440) planes of the spinel ferrite type CoFe_2_O_4_ (JCPDS No. 22-1086), and no impurity peaks were present. A broad peak at around 20–30° was representative for amorphous MCM-41 in the core shell structure. In the diffraction curve of CoFe_2_O_4_/MCM-41 and CoFe_2_O_4_/MCM-41/PA/Cu, the characteristic diffraction peaks were the same as CoFe_2_O_4_, which is a confirmation of the preservation of the phase of this nanoparticle during the functionalization stages. It can be concluded that silica is amorphous and the crystalline structure of CoFe_2_O_4_ magnetic nanoparticles is maintained after silica coating. Crystallite size was determined by the Debye Scherrer's equation for maximum of an observed peak (Table 3). The low XRD profiles of CoFe_2_O_4_/MCM-41 and CoFe_2_O_4_/MCM-41/PA/Cu show the characteristic diffraction peaks of CoFe_2_O_4_ and MCM-41.

**Table 3:** Structural and magnetic properties of the samples synthesized in this study^a^.

Entry	Catalyst	Structural properties			Magnetic properties^e^		
		Size^b^ (nm)	Size^c^ (nm)	Size^d^ (nm)	H_c_ (kOe)	M_r_ (emu/g)	M_s_ (emu/g)
1	MCM-41	–	140–180	–	–	–	–
2	CoFe_2_O_4_	18	15–20	–	1.2	25.6	49.4
3	CF/M	15	140–180 (15–20)	–	0.8	10.6	26.5
4	CF/M//PA/C	15	140–180 (20–25)	150 (20)	0.7	5.7	13.8

^a^Here, CF/M//PA/C denotes catalysts with CoFe_2_O_4_ (CF), MCM-41 (M), N-phenyl anthranilic acid (PA), and Cu (C).

^b^Measured using maximum of an observed peak and Scherrer equation.

^c^Measured using SEM.

^d^Measured using HRTEM.

^e^Here, H_c_, M_r_, and M_s_ denote the values of coercivity, remnant magnetization, saturation magnetization respectively.

#### FE-SEM, EDX, and HRTEM analysis

**Figure 3:**
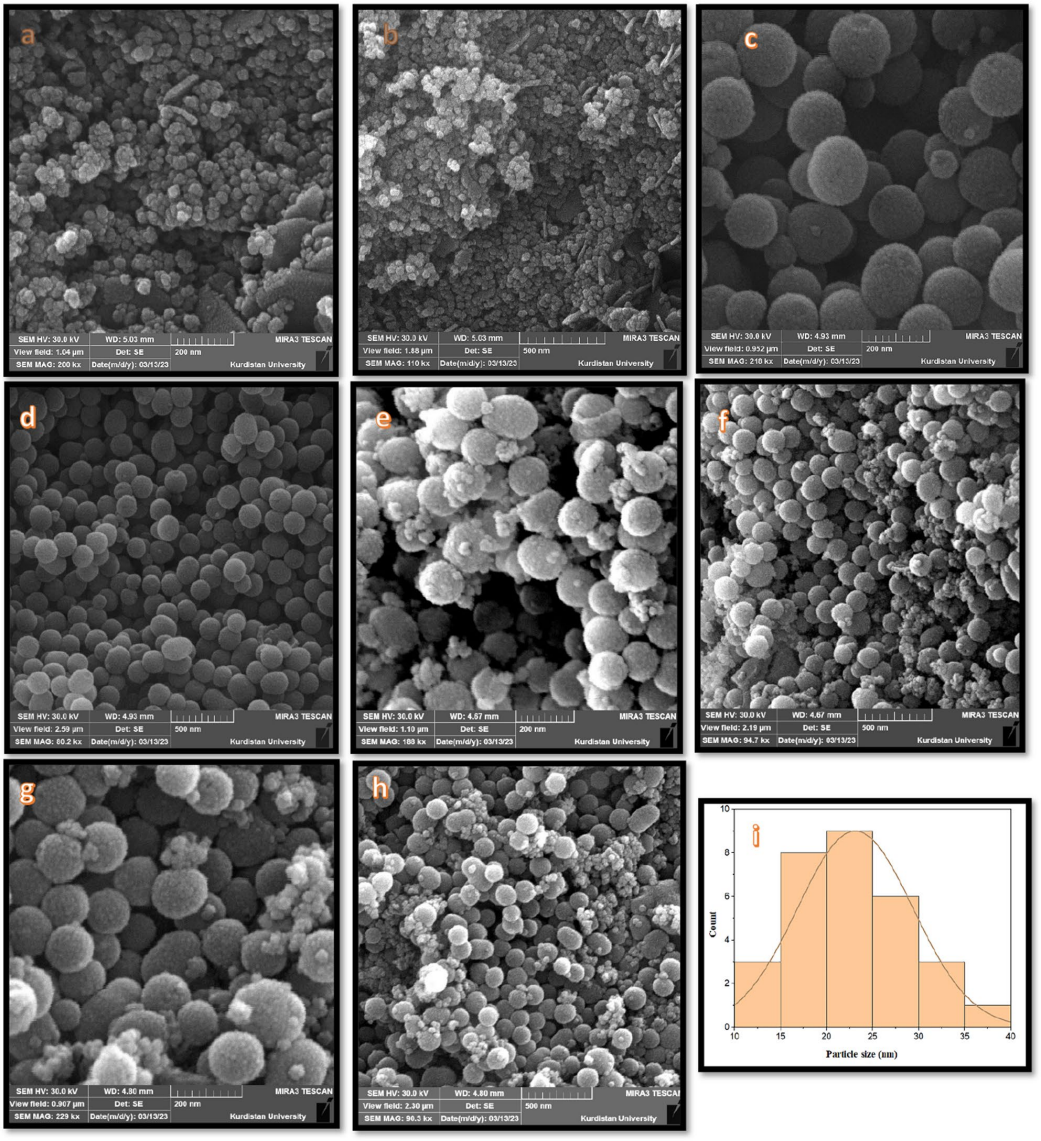
SEM micrographs, and particle size distributions of (**a**,**b**) CoFe_2_O_4_, (**c**,**d**) MCM-41, (**e**,**f**) CF/M, and (**g**,**i**) CF/M/PA/C denotes nanoalloy with CF (CoFe_2_O_4_), M (MCM-41), N-phenyl anthranilic acid (PA), and Cu (C).

**Figure 4:**
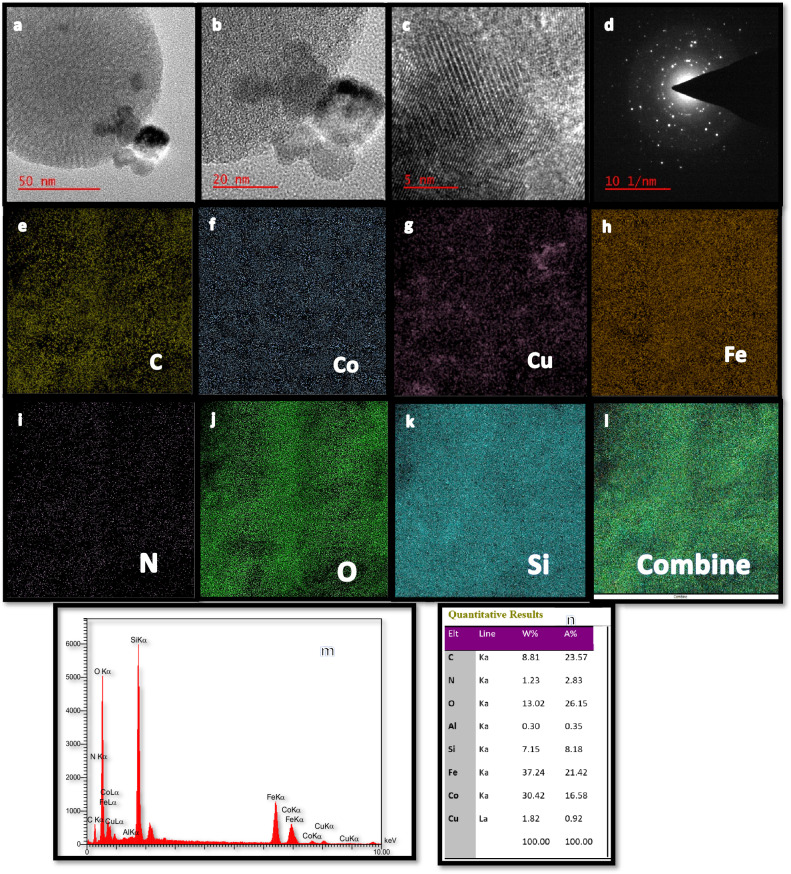
HRTEM micrographs (**a**–**c**), the SAED pattern (**d**), elemental mapping (**e**–**l**), and EDX (**m**, **n**) of CF/M/PA/C denotes nanoalloy with CF (CoFe_2_O_4_), M (MCM-41), N-phenyl anthranilic acid (PA), and Cu (C).

The surface morphology and elemental distribution studies of the MCM-41, CoFe_2_O_4_, CoFe_2_O_4_/MCM-41 and CoFe_2_O_4_/MCM-41/PA/Cu were analyzed by FE-SEM micrographs (Fig. 3), high-resolution HRTEM images (Fig. 4), and EDS elemental mapping (Fig. 4). The FE-SEM images of MCM-41 showed that the particles have a spherical shape with an average diameter of 140–180 nm. The well-ordering and uniformity of the particles with the smooth surface can be observed in this Figure. The FE-SEM images of CoFe_2_O_4_ shows several tiny CoFe_2_O_4_ particles. That the nanoparticles are slightly agglomerated well-distributed spherical particles and an average diameter of 15–20 nm. The FE-SEM images of CoFe_2_O_4_/MCM-41 and CoFe_2_O_4_/MCM-41/PA/Cu confirm the formation of CoFe_2_O_4_/MCM-41 composites due to the presence of particles of MCM-41 and nanoparticles of CoFe_2_O_4_ in the Figure and rough surface with an average particles size of 15–20 nm and 20–25 nm respectively (Table 3). The histogram curve for the size of the CoFe_2_O_4_/MCM-41/PA/Cu were further investigated using SEM (Fig. 3). From this histogram, the particle size of the CoFe_2_O_4_/MCM-41/PA/Cu sample is estimated to be 20–25 nm.

According to HRTEM image the CoFe_2_O_4_/MCM-41/PA/Cu exhibited nearly spherical morphology with black core (CoFe_2_O_4_) and a gray shell (MCM-41), thus suggesting that the CoFe_2_O_4_ magnetite are also homogeneously dispersed inside the MCM-41 silica. HRTEM images of the CoFe_2_O_4_/MCM-41/PA/Cu show pore wall region. In the SAED (selected area electron diffraction) pattern, circularly arranged diffraction spots are clearly seen, indicating the single-phase polycrystalline structure of CoFe_2_O_4_/MCM-41/PA/Cu. Table 3 show the particles size of CoFe_2_O_4_/MCM-41/PA/Cu determined with HRTEM. The EDS spectra and elemental mapping were performed to map the presence of elementals and determine as a quantitative elemental analysis. For CoFe_2_O_4_/MCM-41/PA/Cu shows the Si, Fe, Co, N, C, O and Cu elements.

#### Magnetic properties

**Figure 5:**
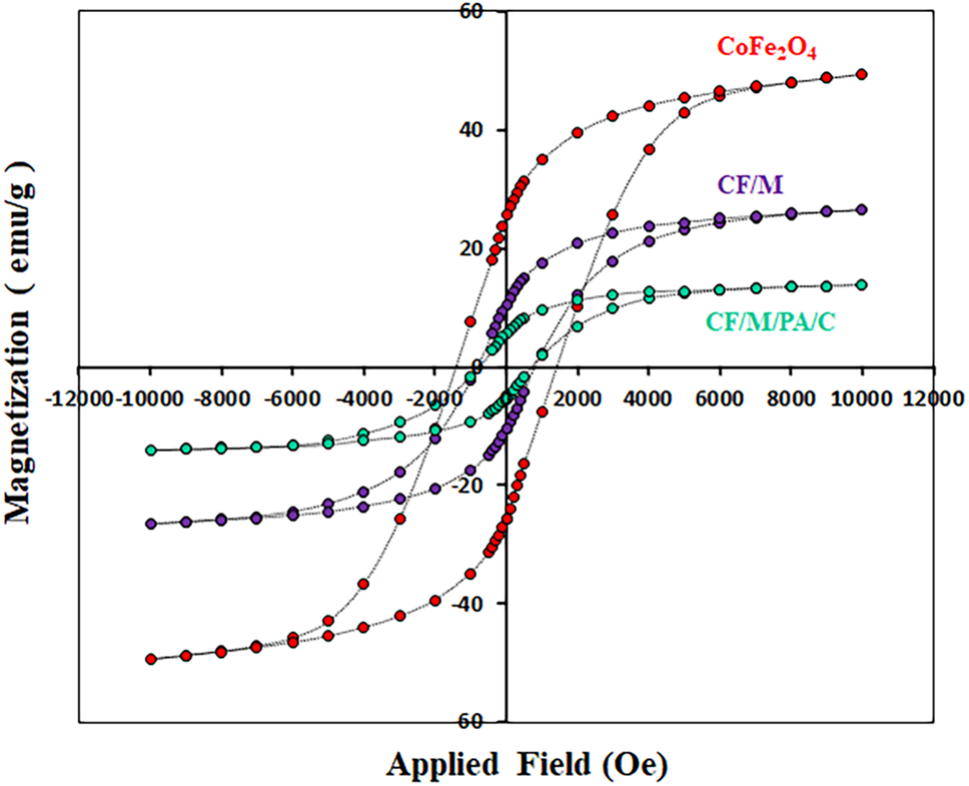
Magnetization curves of CoFe_2_O_4_, CF/M, and CF/M/PA/C denotes nanoalloy with CF (CoFe_2_O_4_), M (MCM-41), N-phenyl anthranilic acid (PA), and Cu (C).

The magnetic field dependence of magnetization of CoFe_2_O_4_, CoFe_2_O_4_/MCM-41 and CoFe_2_O_4_/MCM-41/PA/Cu were measured by a vibrating sample magnetometer (VSM) and the results are presented in Fig. 5. When the applied magnetic field is increased to 15,000 Oe, the magnetization of the samples would approach saturation. The ferromagnetic behavior of the core–shell composites being approved with significant hysteresis loops in the M–H curve. The ferromagnetic behavior confirms that samples can be separated with an applied magnetic field or a magnet. The values of saturation magnetization (Ms), remnant magnetization (Mr), and coercivity (Hc), are given in Table 3. CoFe_2_O_4_, CoFe_2_O_4_/MCM-41 and CoFe_2_O_4_/MCM-41/PA/Cu composites possessed a typical ferromagnetic hysteresis, with a saturation magnetization of 49.41 emu/g and 26.52 emu/g, 13.81 emu/g respectively. The CoFe_2_O_4_ has higher magnetic saturation than those of CoFe_2_O_4_/MCM-41 and CoFe_2_O_4_/MCM-41/PA/Cu, mainly attributing to the non-magnetic coating layer on the surface due to quenching of the surface moment.

#### N_2_ adsorption–desorption isotherms

**Figure 6:**
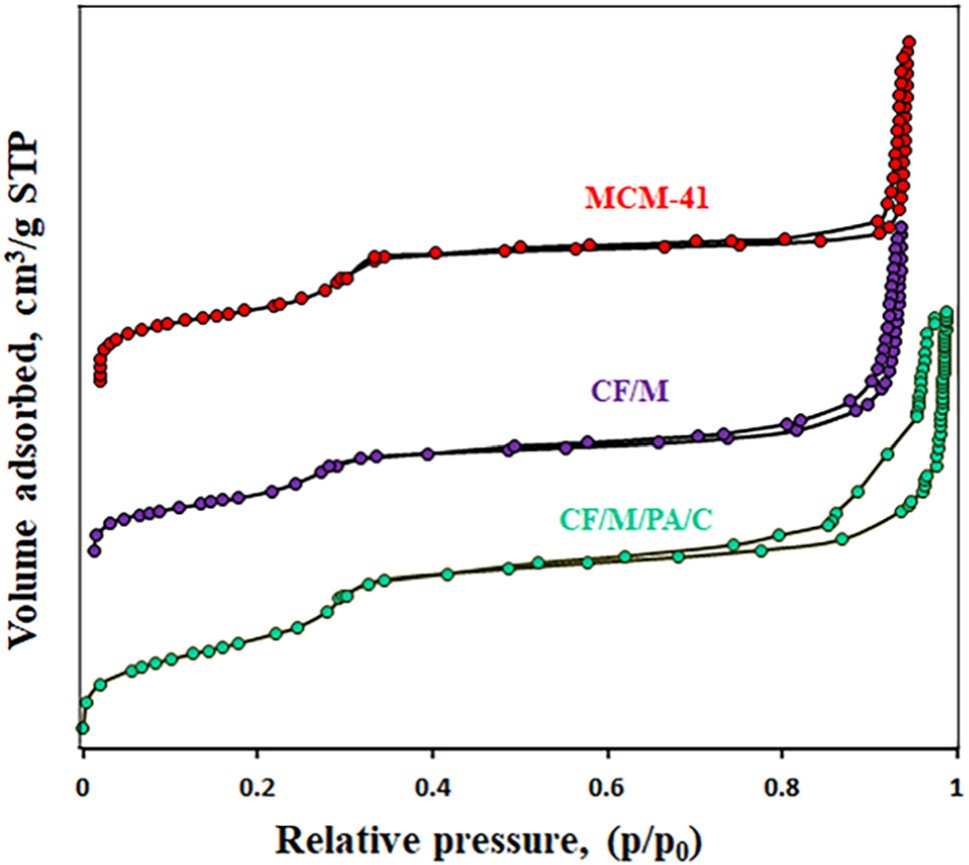
N_2_ adsorption –desorption isotherms of MCM-41, CF/M, and CF/M/PA/C denotes nanoalloy with CF (CoFe_2_O_4_), M (MCM-41), N-phenyl anthranilic acid (PA), and Cu (C).

**Figure 7:**
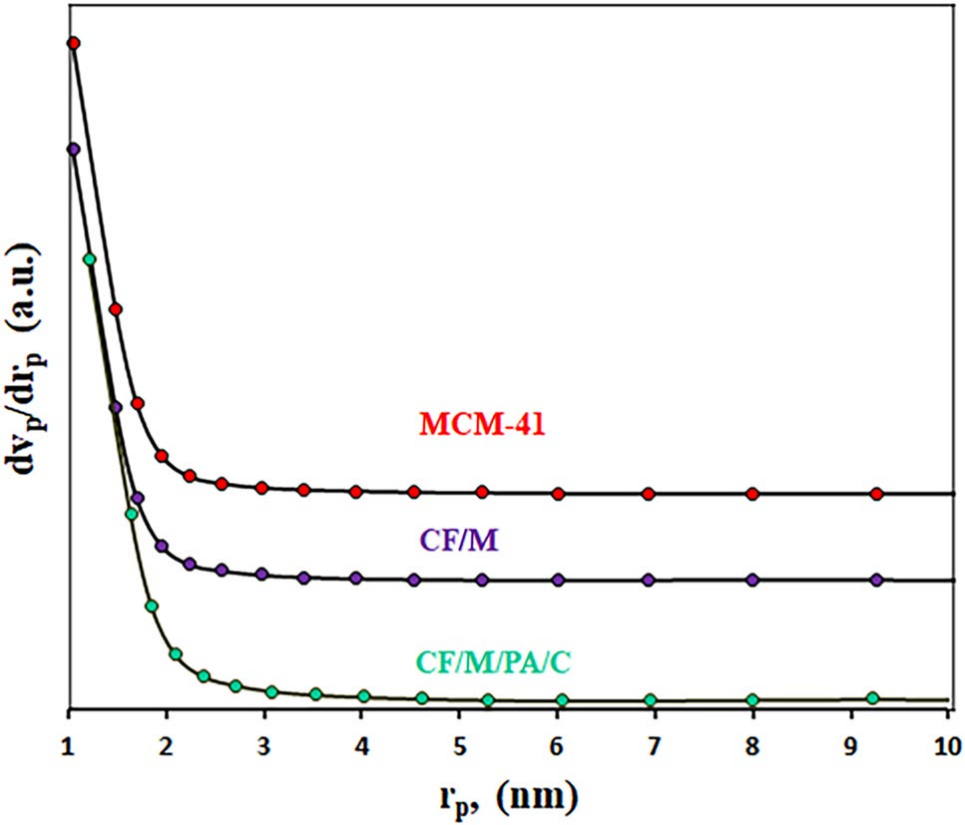
BJH pore width distribution of MCM-41, CF/M, and CF/M/PA/C denotes nanoalloy with CF (CoFe_2_O_4_), M (MCM-41), N-phenyl anthranilic acid (PA), and Cu (C).

The results of N_2_ adsorption–desorption isotherms and the BJH pore size distribution of the MCM-41, CoFe_2_O_4_/MCM-41 and CoFe_2_O_4_/MCM-41/PA/Cu catalyst is illustrated in Figs. 6 and 7 and Table 4. For All the as-synthesized samples, the isotherm curves could be attributed to a type IV adsorption isotherm with a narrow hysteresis loop, as per the IUPAC nomenclature, the prepared composite still preserved mesoporous structure. These results consistently identify type IV isotherms with H1 hysteresis loops for the ordered silicas^45^. Table 4 shows the values of the surface area got by the Brunauer –Emmett –Teller (BET) and t-plot method, pore sizes and pore volumes of MCM-41, CoFe_2_O_4_/MCM-41 and CoFe_2_O_4_/MCM-41/PA/Cu. The MCM-41 shows high surface area by BET and t-plot of 1030.6 m^2^/g and 954.3 m^2^/g respectively, mean pore size of 6.95 nm, and pore volume of 1.79 cm^3^/g. The CoFe_2_O_4_/MCM-41 shows surface area by BET and t-plot of 444.7 m^2^/g and 404.4 m^2^/g respectively, mean pore size of 6.89 nm, and pore volume of 0.875 cm^3^/g. The CoFe_2_O_4_/MCM-41/PA/Cu shows surface area by BET and t-plot of 437.2 m^2^/g and 395.4 m^2^/g respectively, mean pore size of 6.60 nm, and pore volume of 0.721 cm^3^/g. The MCM-41 has higher pore diameter, pore volume, and surface area, than those of CoFe_2_O_4_/MCM-41 and CoFe_2_O_4_/MCM-41/PA/Cu. These results are the indication to the fact that high surface area of MCM-41 could enable the uniform distribution of CoFe_2_O_4_ and PA/Cu and block the pores of MCM-41 but the immobilized group still reveal typical mesoporous structure, which would ensure efficient distribution of catalytic site. Also, the BJH pore size distribution was between 1 and 2 nm for the samples.

**Table 4:** Textural properties of the samples synthesized in this study^a^.

Entry	Catalyst^b^	S_BET_ (m^2^/g)	S _t-Plot_ (m^2^/g)	d_avg_ (nm)	V _total_ (cm^3^/g)
1	MCM-41	1030.6	954.3	6.95	1.790
3	CF/M	444.7	404.4	6.89	0.875
4	CF/M//PA/C	437.2	395.4	6.60	0.721

^a^Here, S_BET_, S _t-Plot_, d_avg_, and V_total_ denote the Brunauer –Emmet –Teller specific surface area, surface area by t-plot, average pore size, and total pore volume respectively.

^b^Here, CF/M//PA/C denotes catalysts with MCM-41 (M), CoFe_2_O_4_ (CF), N-phenyl anthranilic acid (PA), and Cu (C).

#### FT-IR analysis

**Figure 8:**
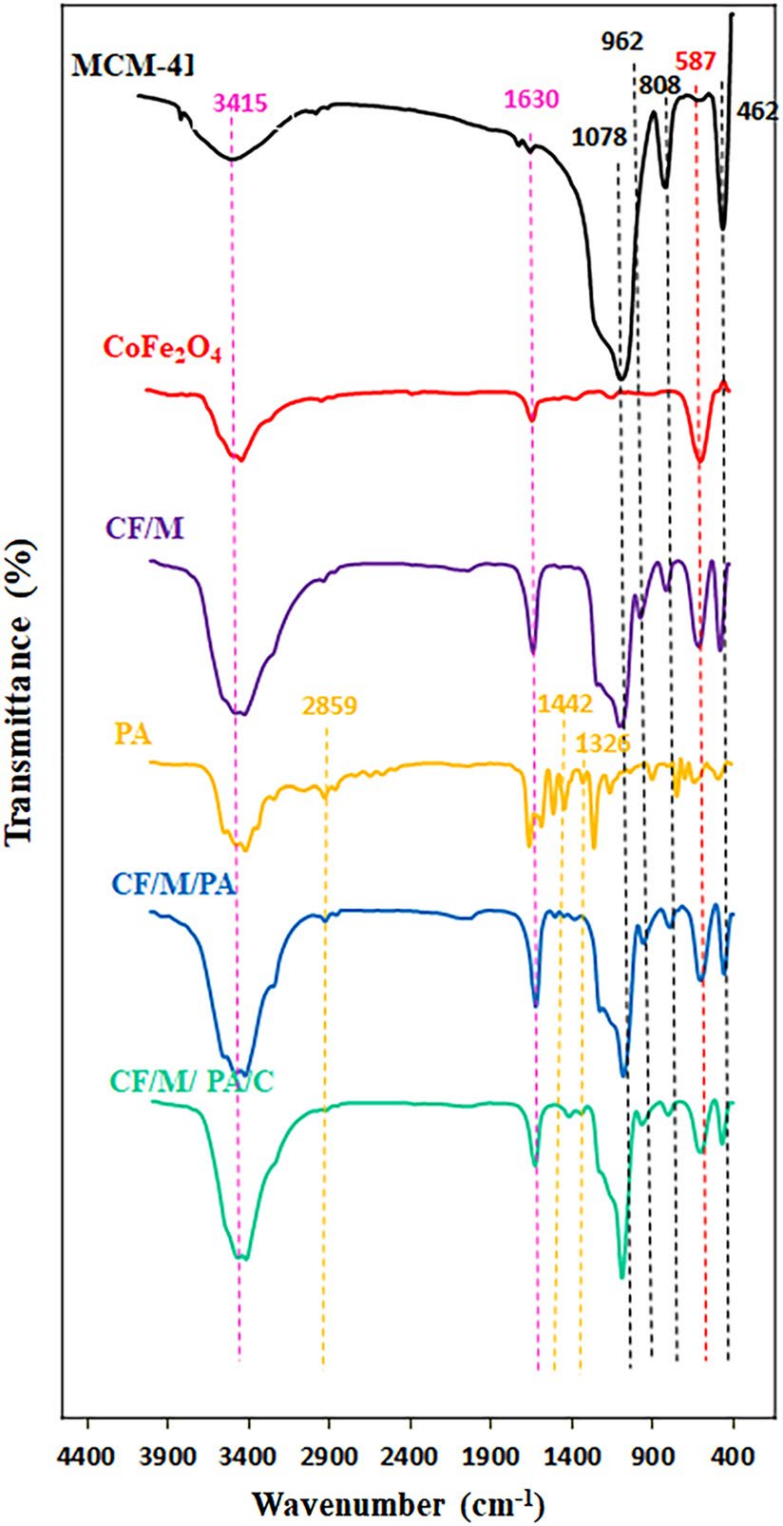
FT-IR spectra of MCM-41, CoFe_2_O_4_ CF/M, PA, CF/M/PA and CF/M/PA/C denotes nanoalloy with CF (CoFe_2_O_4_), M (MCM-41), N-phenyl anthranilic acid (PA), and Cu (C).

FT-IR spectrum related to MCM-41, CoFe_2_O_4_, CoFe_2_O_4_/MCM-41, PA and CoFe_2_O_4_/MCM-41/PA, and CoFe_2_O_4_/MCM-41/PA/Cu catalyst can be seen in Fig. 8. The sharp bands associated with OH stretching vibrations of surface hydroxyl groups and water presented at 3200 and 3700 cm^−1^. A medium band in the region of 1623–1650 cm^−1^ is mainly attributed to the H–O–H bending motion. From Fig. 1, the signals observed at round 462 cm^−1^ can be correlated with the rocking motion of bridging oxygens band in Si–O–Si. In addition, the symmetric and asymmetric stretching vibration of Si–O–Si bonds can be correlated with the 808 cm^−1^ and 1078 cm^−1^ bands respectively which is common to all the spectra. The extra bands at 960–970 cm^−1^ are attributed to incorporation of metal into the skeleton of mesoporous MCM-41 sample. This band is mainly attributed to the vibration mode of Si–OH, but the intensity of the band increases and is shifted to lower wavenumber when metals are attached. It indicates the incorporation of MCM-41 silica onto the magnetite nanoparticles. In all curves, the signals observed at round 587 cm^−1^ can be assigned to the stretching modes of Fe–O bond. The special bands in the FT-IR of the PA at around 1326 cm^−1^, 1442 cm^−1^, and 2859 cm^−1^ can be attributed to the stretching vibration of C–N of amine, the C–H bending, and the CH_2_ symmetric stretching vibration respectively. After modification of the PA, the specific bands are observed in the FT-IR of the CoFe_2_O_4_/MCM-41/PA. The presence of Lewis and Brønsted acid sites indicating the acidic characteristics of catalysts was determined with DRIFT spectra by Şimşek and et al.^46^. In this paper, the Lewis acid site of catalysts was identified with the FT-IR. The FT-IR spectrum of the CoFe_2_O_4_/MCM-41/PA/C sample (Fig. 8) shows special bands of the FT-IR of the PA, which has been shifted to a lower wavenumber, showing that PA ligand are coordinated to Lewis’s acid site (Cu (II)).

### Catalyst evaluation

The catalyst was investigated in the role of the catalysts for the one-pot conversion of nitrile to tetrazole with 4-chlorobenzonitrile (1 mmol), catalyst (100 mg), NaN_3_ (1.2 mmol), at 100 °C in an aqueous medium, and the results are listed in Table 5. In the presence of the MCM-41 and CoFe_2_O_4_ support without any metal loading, nitrile was not converted (Table 5, entry 1 and 2). The nitrile conversion over the CoFe_2_O_4_/MCM-41 was 30% (Table 5, entry 3), which indicated the important of composite. When the nitrile conversion reaction was performed over the CoFe_2_O_4_/MCM-41/PA product was moderate (60%) but the tetrazole selectivity was 100% (Table 5, entry 4). The nitrile conversion over the CoFe_2_O_4_/MCM-41/PA/Cu catalyst was high (84%; Table 5, entry 5), which indicated the important role of the Cu metal sites for the production of tetrazole, the high Lewis acidity of the catalyst can facilitate the adsorption of substrate. To elucidate the significant activity of CoFe_2_O_4_/MCM-41/PA/Cu catalyst, CoFe_2_O_4_/PA/Cu catalyst was prepared, and catalytic performance was compared with that of the CoFe_2_O_4_/PA/Cu catalyst. According to Table 5, CoFe_2_O_4_/MCM-41/PA/Cu catalyst has very high activity compared to CoFe_2_O_4_/PA/Cu.

**Table 5:** 4-Chlorobenzonitrile conversion, product selectivity, yields, TON, and TOF over several catalysts^a^.

Entry	Catalyst	T^b^	C (%)^c^	S (%)^d^	Y^e^ (%)	TN^f^	TF^g^
1	MCM-41	30	0	100	0	0	0
2	CoFe_2_O_4_	30	0	100	0	0	0
3	CF/M	30	30	100	30	4.28	8.56
4	CF/M/PA	30	60	100	60	8.57	17.14
5	CF/M/PA/C	10	88	100	88	12.06	72.65
6	CF/PA/C	30	50	100	50	7.14	14.28

Here, CF/M/PA/C denotes catalysts with CoFe_2_O_4_ (CF), MCM-41 (M), N-phenyl anthranilic acid (PA), and Cu (C).

^a^Reaction conditions: Nitrile (1 mmol), NaN_3_, (1.2 mmol), catalyst (100 mg) and water (3mL) at 100 °C. ^b^Time (min).

^c^Conversion of nitril. ^d^Product Selectivity. ^e^Isolated yield. ^f^TON. ^g^TOF.

**Figure 9:**
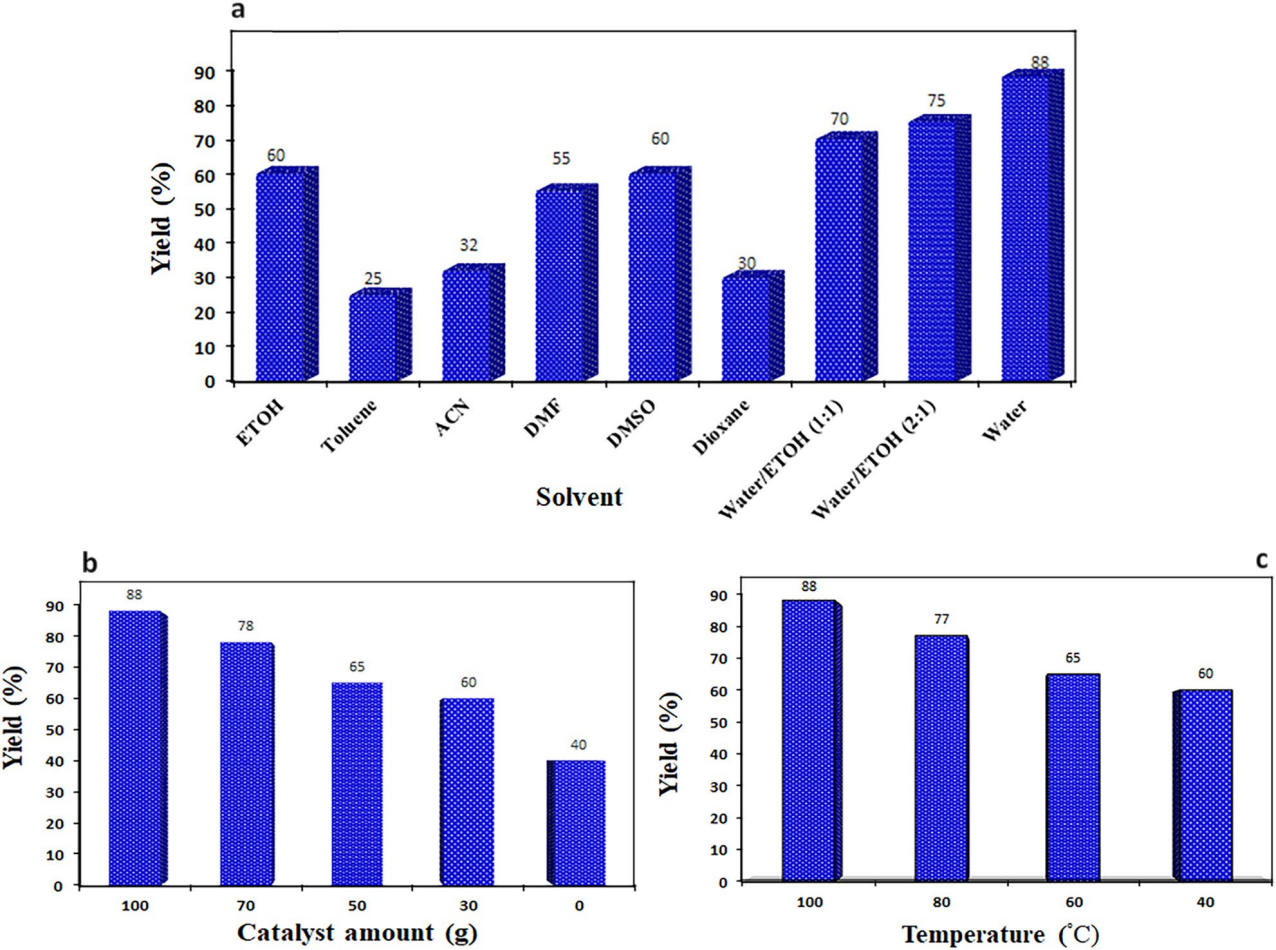
5-(4-Chlorophenyl))-1*H*-tetrazole yield at different (**a**) solvent (**b**) catalyst amount, and (**c**) temperatures during one hour.

The catalytic efficiency of the designed CoFe_2_O_4_/MCM-41/PA/Cu composite was examined in the synthesis of tetrazole. To test the activity of the catalyst the reaction of 4-chlorobenzonitrile (1 mmol), with NaN_3_ (1.2 mmol) was selected as model substrates and the influences of some parameters on the synthesis of tetrazole including; temperatures, amount of catalyst, and solvents were investigated using CoFe_2_O_4_/MCM-41/PA/Cu as the catalyst during one hour (Fig. 9). The reaction was screened with various solvents such as EtOH, DMSO, DMF, dioxane, acetonitrile, EtOH/water, toluene, and water were tested. The use of a protic polar solvent will rise the reaction rate. To develop an environmentally benign, the use of nontoxic and inexpensive solvents should be considered. In this work, in water the highest yield is observed. The other solvents, had lower yields.

We have also studied the effect of catalyst weights (0, 30, 50, 70 and 100 mg). The results displayed that the progress of the yield was raised from 60 (30 mg) to 65 (50 mg) then to 78 (70 mg) and 88% (100 mg). The results displays that the best yield was achieved at 100 mg of the catalyst and the product yield was increased with small amount after 100 mg.

We have also carried out the reaction in different temperature. The results displayed that the progress of the yield was raised from 60 (40 °C) to 65 (60 °C) then to 77 (80 °C) and 88% (100 °C). The best result was obtained at 100 °C.

After optimization of the reaction conditions, we extended the scope of the reaction for various derivatives of nitrile compounds. Accordingly, both electron-donating and electron withdrawing substituents have been employed for this reaction. The conversion could also be very successfully carried out at very short time. These results strongly confirm that this method is applicable for green and fast conversion of a large variety of derivatives that is an excellent advantage in the green chemistry world (Table 6).

**Table 6:** Synthesis of 5-substituted 1*H*-tetrazoles derivatives in the presence of CF/M/PA/C.

Entry	Substrate	Time (min)	Yield (%)	TON	TOF (h^−1^)	M. p (°C)
1	3-Nitrobenzonitrile	30	89	12.71	25.42	143–145
2	4-Nitrobenzonitrile	20	87	12.34	37.06	215–218
3	3-Bromobenzonitrile	16	85	12.06	45.34	180–183
4	4-Bromobenzonitrile	10	90	13.01	78.37	262–265
5	Benzonitrile	20	91	13.01	39.00	212–215
6	2-Phenylacetonitrile	15	85	12.14	60.7	122–124
7	4-Hydroxybenzonitrile	18	89	12.70	42.33	235–236
8	2-Chlorobenzonitrile	24	88	12.57	31.43	175–179
9	3-Chlorobenzonitrile	20	88	12.57	37.71	261–264
10	4-Chlorobenzonitrile	10	88	12.57	72.65	131–133

Temperature = refluxing conditions; Nitrile = 1 mmol; NaN_3_ = 1.2 mmol; Catalyst amount = 0.1 g; H_2_O = 3 mL.

**Figure 10:**
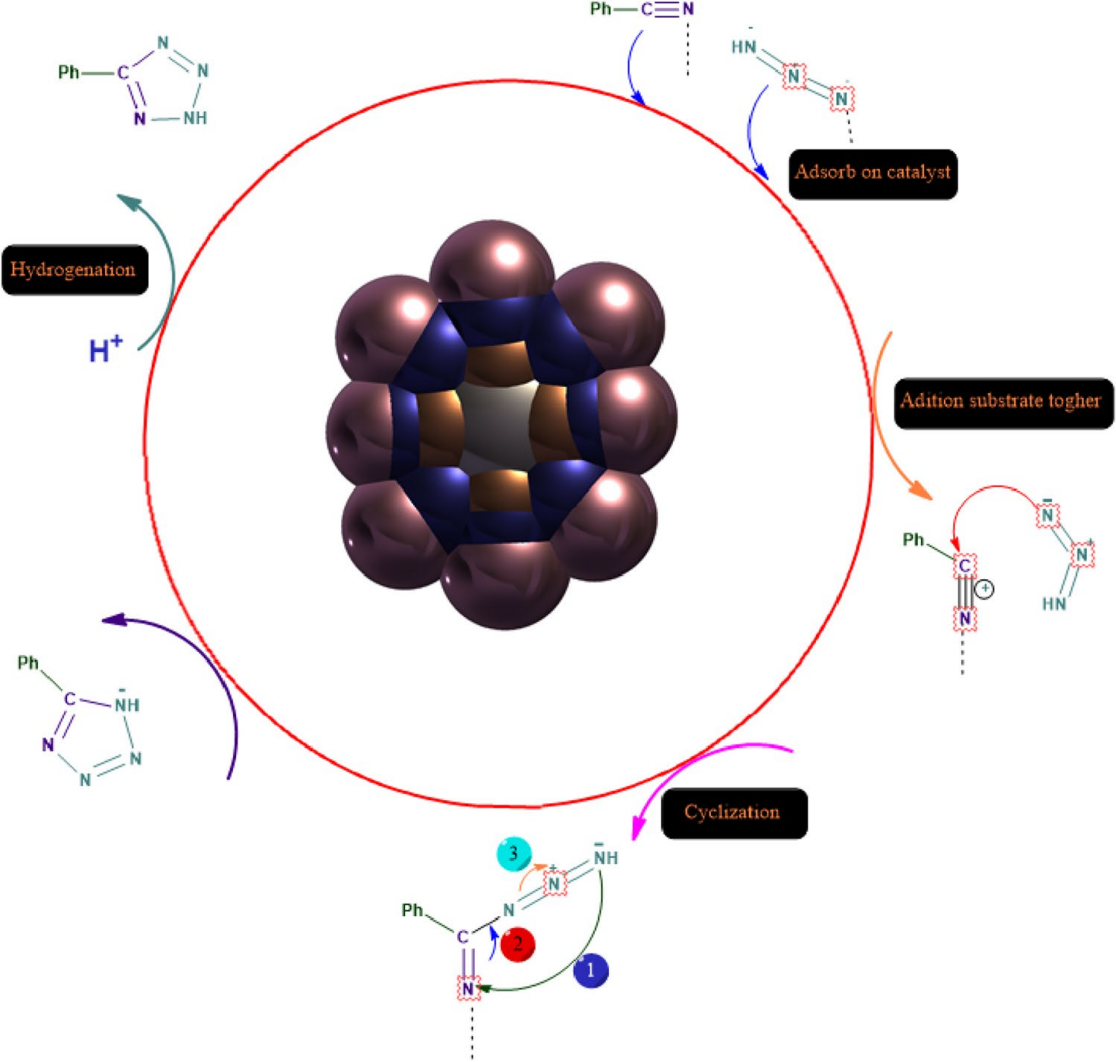
Plausible Reaction Pathway for the Direct Conversion of over the CoFe_2_O_4_/MCM-41/PA/Cu Catalyst.

The source of the catalysis in the formation of 1*H*-tetrazoles is the coordination of the nitrile substrate to the Lewis acidic copper. The dominant factor influencing [2 + 3] cycloaddition is coordination of Cu^2+^ to the nitrile, and. Subsequent nucleophilic attack by azide, followed by hydrolysis produces tetrazole as the end product^47^ (Fig. 10).

### Catalyst reusability

**Figure 11:**
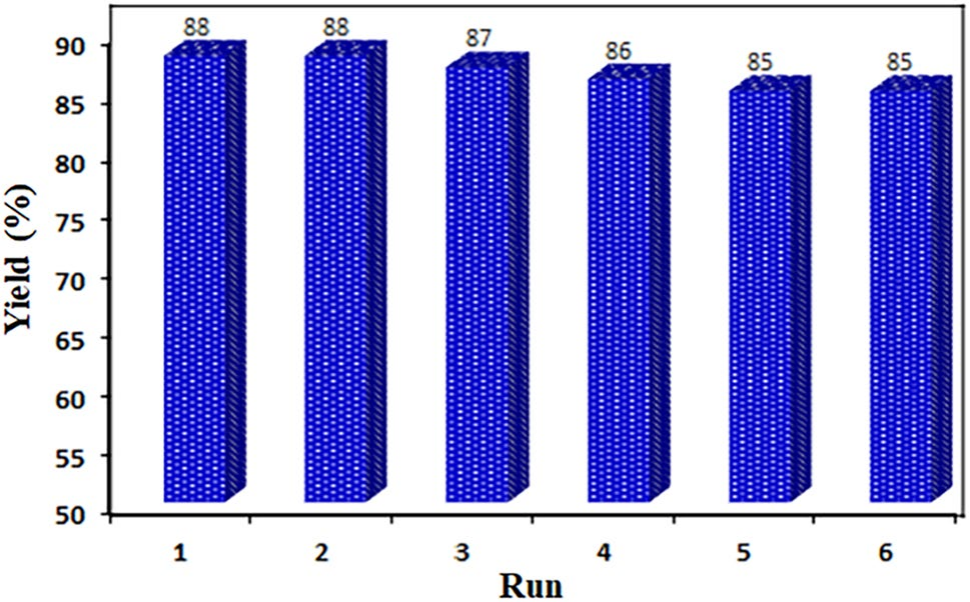
Reusability of the CoFe_2_O_4_/MCM-41/PA/Cu catalyst. Reaction conditions: 4-chlorobenzonitrile (1 mmol), NaN_3_, (1.2 mmol), catalyst (100 mg) and water (3mL) at 100 °C.

The reusability of the designed catalyst was studied for the one-pot conversion of nitrile to tetrazole with 4-chlorobenzonitrile (1 mmol), catalyst (100 mg), NaN_3_ (1.2 mmol), at 100 °C in an aqueous medium and the findings are given in Fig. 11. For this, after the reaction was completed, the spent catalyst was removed after each run through applying an external magnet, then washed with ethyl acetate and used for the next run. The catalyst showed nearly the same performance compared with the first run even after six consecutive reaction runs in terms of the conversion of nitrile. The stable performance could be related to the negligible leach in the Cu loading. In another study a leaching test was performed to show the nature of catalyst in the reaction process. To do this, the reaction was stopped after about 50% of the reaction process was completed, then the catalyst was removed using an external magnet. The catalyst-free mixture was then allowed to continue for 20 min. In this case no further conversion was observed indicating that the catalyst operates in a heterogeneous manner.

## Conclusion

The present work successfully developed a novel catalyst with core –shell structure (CoFe_2_O_4_/MCM-41/PA/Cu) for the one-pot the conversion of nitrile to tetrazole that has powerful catalytic activity, high separation efficiency and good reusability. The incorporation of CoFe_2_O_4_ could not only impart the catalyst with a strong magnetism, but also tune its acidity to promote tetrazole production. A high yield was achieved from nitrile conversion in an aqueous medium. The as- catalyst could be reused at least six times by an external magnetic field without substantial change in catalytic activity. This proposed strategy exposes some advantages over those available towards tetrazole production from nitrile, such as the nonuse of hazardous metal or dual catalysts, fine catalyst recyclability with magnetic separation, and the implementation of a green and sustainable route.

### Supplementary Information

Supplementary Information.

## Data Availability

The datasets used and/or analyzed during the current study available from the corresponding author on reasonable request.
