# Supplementary material for: Copper-decorated core–shell structured ordered mesoporous containing cobalt ferrite nanoparticles as high-performance heterogeneous catalyst toward synthesis of tetrazole
Source: Sci Rep. 2023 Sep 13;13:15146. doi: 10.1038/s41598-023-42094-1 (PMC10499787; doi:10.1038/s41598-023-42094-1)
Supplement: Supplementary file 1 — Supplementary Information. [file 41598_2023_42094_MOESM1_ESM.docx]

Supporting Information

**Copper-Decorated** **Core–Shell Structured** **Ordered Mesoporous Containing Cobalt Ferrite Nanoparticles as High-Performance Heterogeneous Catalyst toward Synthesis of Tetrazole**

Somayeh Molaei, Mohammad Ghadermazi^^[[1]](#footnote-1)^*^

University of Kurdistan, Department of Chemistry, Faculty of Science, Sanandaj, Iran

1. Experimental

1.1 Materials

Copper nitrate three hydrate (Cu (NO_3_)_2_∙3H_2_O), N-phenyl anthranilic acid (PA), iron (III) chloride hexahydrate (FeCl_3_∙6H_2_O), Cobalt (II) chloride hexahydrate (CoCl_2_∙6H_2_O), Sodium hydroxide (NaOH), Dodecyltrimethylammonium bromide (Dctab), tetraethyl orthosilicate (TEOS), were purchased from Sigma-Aldrich. Ethanol (ETOH), 1,4 dioxane, dimethyl sulfoxide (DMSO), Toluene, Dimethylformamide (DMF), and Acetonitrile (ACN) were purchased from Merck without further purification.

1.2 Synthesis of the MCM-41

In order to synthesize MCM-41 mesopores with sol gel method, 1 g of Dctab surfactant was added to a solution containing 480 mL of deionized water and 3.5 mL of 2 M sodium hydroxide at a temperature of 80 °C with stirring. After the mixture became clear and homogenous, 5 mL of TEOS was slowly added drop by drop as a source of silica, and the reaction mixture was stirred for 2 hours at 80 °C. After the reaction time, the mixture was cooled to room temperature. Then the mixture was filtered and washed several times with deionized water. The white solid powder was dried in an oven at 70 °C. Finally, the white powder was placed in the oven for 5 hours at a heating rate of 2 °C/min to a temperature of 550 °C to calcine and remove the surfactant [1].

1.3 Synthesis of the CoFe_2_O_4_ nanoparticles

To synthesize CoFe_2_O_4_ nanoparticles with coprecipitation method, in a 100 ml round-bottom flask, 1 mmol of FeCl_3_∙6H_2_O powder and 0.5 mmol of CoCl_2_∙2H_2_O powder were dissolved in 50 mL of distilled water that had been previously deaerated with nitrogen gas. Then 1 g of NaOH was dissolved in 25 mL of distilled water and added drop by drop to the initial solution. The final solution was refluxed for 3 hours. Then the reaction was cooled to ambient temperature and the black precipitate formed was separated using an external magnet, and after washing with ethanol, the obtained CoFe_2_O_4_ nanoparticles were dried at 61°C [2].

1.4 Synthesis of the CoFe_2_O_4_ /MCM-41 nanocomposite

To synthesize CoFe_2_O_4_ /MCM-41 nanocomposite with wet impregnation method, 2.0 g CoFe_2_O_4_ nanoparticles was added to a mixture of deionized water and ethanol and (10 mL deionized water/70 mL ethanol). After dispersing the solution with sonication for 20 min, 5 mL aqueous ammonia and 1.72 g MCM-41 were added. The mixture was stirred for another 24 h) at room temperature (. The obtained CoFe_2_O_4_ /MCM-41 nanocomposite was separated using an external magnet, rinsed with deionized water and ethanol then the nanocomposite was dried at 80 °C under vacuum for 12 h.

1.5 Synthesis of the CoFe_2_O_4_ /MCM-41/PA nanocomposite

To synthesize CoFe_2_O_4_ /MCM-41/PA nanocomposite with post-functionalization modification method, a mixture containing 1 g CoFe_2_O_4_ /MCM-41 and 1.5 g of N-phenyl anthranilic acid (PA) ligand was refluxed in water solvent for 48 h. After the completion of the reaction, the reaction product (CoFe_2_O_4_/MCM-41/PA) was separated with a magnet and dried after washing with water and ethanol.

1.6 Synthesis of the CoFe_2_O_4_ /MCM-41/PA/Cu nanocomposite

To synthesize CoFe_2_O_4_/MCM-41/PA/Cu nanocomposite with post-functionalization modification method, 1 g CoFe_2_O_4_ /MCM-41/PA with 1.5 g Cu (NO_3_)_2_∙3H_2_O were mixed in 30 mL ethanol solvent and the reaction mixture was refluxed for 24 h.

1.7 Catalyst characterization

The devices used to identify the synthesized products and catalysts have the following specifications: Fourier Transform Infrared (FT-IR) spectrophotometry was carried out on a Bruker VRTEX 80 v instrument. Field emission scanning electron microscopy (FESEM) was carried out on FESEM-TESCAN MIRA3 equipment. X-Ray diffraction (XRD) was performed on a Scintag PAD V X-Ray diffractometer using a Cu (Kα = 1.54 Å). Nitrogen adsorption-desorption isotherm was carried out on Volumetric adsorption analyzer Japan, Belsorp-mini II, using a N_2_ gas manifold at 77 K. High-resolution transmission scanning electron microscope (HR-TEM) was carried out on a Zeiss-EM10C instrument. Melting points was carried out on an Electrothermal 9100 apparatus. Nuclear magnetic resonance (NMR) spectra were performed on a Bruker (^1^H at 400 MHz) instrument.

1.5. General process for the synthesis of 1*H*- tetrazoles

3 mL of H_2_O was added to the mixture of nitrile (1 mmol), sodium azide (1.2 mmol), and catalyst (70 mg) and placed at 80 ^°^C. Progression of the reaction was followed by TLC. After the end of the reaction, the catalyst was filtered and the reaction mixture was treated with ethyl acetate and acidified with HCl (10 mL, 5 N). After that, 2-5 mL of cold water was added and washed several times. Finally, it was dried.

1.6. Spectral data

5-(3-Nitrophenyl)-1*H*-tetrazole

(Table 3, Entry1): ^1^H­NMR (400 MHz, DMSO, ppm): δ 7.95-7.97 (m, 1H), 8.46–8.53 (m, 2H), 8.88 (s, 1H).

5-(4-Nitrophenyl)-1*H*-tetrazole

(Table 3, Entry 2): ^1^H­NMR (400 MHz, DMSO, ppm): δ 8.37–8.44 (d, 2H), 8.49–8.50 (d, 2H).

5-(4-Bromophenyl))-1*H*-tetrazole

(Table 3, Entry 14): ^1^H­NMR (400 MHz, DMSO, ppm): δ 7.86–7.88 (m, 4H).

| 𝐶𝑜𝑛𝑣𝑒𝑟𝑠𝑖𝑜𝑛 (%) = ( $\frac{moles of \mathrm{converted}\mathrm{reactant}}{initial moles of reactant}$) × 100 |
| --- |
| 𝑌𝑖𝑒𝑙𝑑 (%) = ( $\frac{moles of product}{initial moles of reactant}$) × 100  *Selectivity* (%) = $\frac{\mathrm{Yield}}{\mathrm{Conversion}}$× 100 |
| *TON =* $\frac{Number of moles of product}{Number of moles of an active catalyst}$ |
| *TOF* = $\frac{\mathrm{TON}}{Time of reaction}$ |

As the particle size has been estimated by the Sherrer formula (D = 0.94 λ/β cosɵ, where D is the average grain size, λ the X-ray wavelength (0.15406 nm), and ɵ and β the diffraction angle and full-width at half maximum of an observed peak, respectively.(

| Catalyst | K | λ (A֯) | *Peak position 2ɵ* | *Cosɵ* | β= FWHM  (֯) | β= FWHM  Rad | D(nm) |
| --- | --- | --- | --- | --- | --- | --- | --- |
| MCM-41 | 0.94 | 1.5406 | 23.22006 | 0.5765138 | 12.38378 | 0.216137734 | - |
| CoFe_2_O_4_ | 0.94 | 1.5406 | 35.86175 | 0.95142864 | 0.47666 | 0.008319286 | 18.30 |
| M/CF | 0.94 | 1.5406 | 35.64686 | 0.95200430 | 0.56426 | 0.009848194 | 15.4 |
| M/CF/PA/C | 0.94 | 1.5406 | 35.74975 | 0.95172909 | 0.55337 | 0.00965812 | 15.7 |

D= $\frac{0.9 \lambda}{\beta*COS ɵ}$ = $\frac{1.448164}{0.008319286 * 0.95142864}=182.96 A֯=18.30 nm$

[1] S. Molaei, M. Ghadermazi, Micropor. Mesopor. Mat. 319 (2021) 110990

[2] N. Moeini, S. Molaei, M. Ghadermazi, J. Mol. Struct. 1246 (2021) 131071

[3] V. Şimşek, S. Şahin, J. Porous Mater. 26 (2019) 1657-1665

[4] X. Hong, Y. Sun, T. Zhu, Z. Liu, Catal. Sci. Technol. 6 (2016) 3606-3615

1. * Address correspondence to University of Kurdistan, Department of Chemistry, Faculty of Science, Sanandaj, Iran.; e-mail: [mghadermazi@yahoo.com](mailto:mghadermazi@yahoo.com)

   Tel: 989183723970 [↑](#footnote-ref-1)
